# Cross-border mobility responses to COVID-19 in Europe: new evidence from facebook data

**DOI:** 10.1186/s12992-022-00832-6

**Published:** 2022-04-18

**Authors:** Fredérić Docquier, Nicolas Golenvaux, Siegfried Nijssen, Pierre Schaus, Felix Stips

**Affiliations:** 1 Crossing Borders, Luxembourg Institute for Socio-Economic Research (LISER), Esch-sur-Alzette, Luxembourg; 2 Institute for Information and Communication Technologies, Electronics and Applied Mathematics (ICTEAM), Université catholique de Louvain, Louvain-la-Neuve, Belgium

**Keywords:** Cross-border mobility, Covid-19, Containment policies, Non-Parmaceutical interventions

## Abstract

**Background:**

Assessing the impact of government responses to Covid-19 is crucial to contain the pandemic and improve preparedness for future crises. We investigate here the impact of non-pharmaceutical interventions (NPIs) and infection threats on the daily evolution of cross-border movements of people during the Covid-19 pandemic. We use a unique database on Facebook users’ mobility, and rely on regression and machine learning models to identify the role of infection threats and containment policies. Permutation techniques allow us to compare the impact and predictive power of these two categories of variables.

**Results:**

In contrast with studies on within-border mobility, our models point to a stronger importance of containment policies in explaining changes in cross-border traffic as compared with international travel bans and fears of being infected. The latter are proxied by the numbers of Covid-19 cases and deaths at destination. Although the ranking among coercive policies varies across modelling techniques, containment measures in the destination country (such as cancelling of events, restrictions on internal movements and public gatherings), and school closures in the origin country (influencing parental leaves) have the strongest impacts on cross-border movements.

**Conclusion:**

While descriptive in nature, our findings have policy-relevant implications. Cross-border movements of people predominantly consist of labor commuting flows and business travels. These economic and essential flows are marginally influenced by the fear of infection and international travel bans. They are mostly governed by the stringency of internal containment policies and the ability to travel.

## Background

Covid-19 is a disease induced by severe acute respiratory syndrome coronavirus 2 (SARS-CoV-2). The first known cases of Covid-19 were registered in December 2019 in Wuhan (China), but the virus rapidly spread into many countries, leading to a global pandemic that has held the world captive for many months. As until recently no effective vaccine or medication was available, government policies were mainly targeted towards tracing and disrupting infection chains. Many countries introduced coercive measures and disincentives to limit within- and cross-border mobility of people in the hope of reducing the virus propagation. Whether these policies were effective or not remains an open question, and there is much to be gained from better understanding the evolution and determinants of people’s mobility during Covid times.^1^

There is strong evidence that *within-border* (or internal) people’s mobility declined during the Covid-19 crisis. Existing literature relies on big data provided by private cellular phone companies,^2^ and documenting spatial movements in real time. [2] uses Vodafone data for Italy, Portugal and Spain, and finds that women and younger people show the largest drop in mobility. [3] combines telco data with household surveys to highlight a sharp decline in short-distance mobility, as proxied by daily time spent at parks, retail and recreation, grocery, transit locations, and workplaces. Using mobility data from the analytics company SafeGraph, [4] finds that the decline in mobility in New York and in four other U.S. cities is mostly driven by the fear of infection, rather than by legal restrictions. Although it also finds a significant impact of non-pharmaceutical interventions (NPIs) such as closing nonessential businesses, sheltering in place, and school closures, the dominant role of infection threats is confirmed by [5], who relies on Google mobility data.

By contrast, there is scant evidence of the impact of Covid-19 on *cross-border* (or international) mobility, which is due to the absence of high-frequency data on border crossings.^3^ In this paper we aim to fill this research gap by addressing the following research questions: (i) How has the Covid-19 impacted cross-border movements of people? (ii) Are these changes due to coercive measures (such as containment policies or international travel bans) or by the fear of contracting the virus?

Understanding the determinants of cross-border mobility responses to Covid-19 is important for economic and epidemiological reasons. Cross-border movements of people predominantly consists of labor commuting flows and business travels. Economically speaking, labor mobility is a key ingredient for growth and competitiveness in normal times. And in a pandemic context, restrictions placed on how workers move around can slow down economic recovery prospects, by making it more difficult for businesses to hire productive workers. They can also induce severe economic impacts on cross-border workers and their families. Epidemiologically speaking, the role that mobility is playing in the spread of the disease is still unclear. Using SafeGraph data for New York city and for other U.S. cities, [6] find that (internal) mobility increased the spread of the disease in the early stage of the pandemic. [7] also finds a strong association between internal movements and mortality using Google mobility data for the UK. In the same vein, [8] shows that (internal and international) travel bans enacted during the Chinese Lunar New Year holiday helped reduce the spread of the virus, and [9] argue that an appropriate coordination would considerably improve the likelihood of eliminating community transmission throughout Europe. By contrast, others expressed skepticism about the epidemiological consequences of travel bans, arguing that the impacts of these restrictions are not well understood [10] or poorly effective [11–15] once patient zero has already spread the virus across regions.

Without taking any position on the fact that cross-border mobility should be limited or encouraged, we use a unique database on daily mobility of European Facebook users to shed light on the evolution of cross-border movements of people during an entire pandemic year, and to compare the effects of coercive policies with those related to the fear of infection. Our results contrast with those obtained for internal mobility. The following sections successively describe our data sources, methods and findings.

## Data

### Border crossings

Data on daily cross-border mobility is obtained from Facebook (denoted by FB, henceforth) for the period from the 29th of February 2020 to the 28th of February 2021 [16]. The database documents cross-border flows of FB users with *location services enabled*, who travel from an origin to a destination country by any means of transportation (car, train, air, etc.) during each 24-hour time period^4^. Only daily flows with a minimum of 1,000 movers are reported in the dataset. All flows below 1,000 are set to zero in order to minimize re-identification risk of FB users. This means that our outcome variable is left-censored. To limit the impact of censoring, we focus on 45 country pairs (involving 30 contiguous European countries) characterized by at least 25% of uncensored values of daily traffic during the period of observation. This selection limits the ability to generalize our results but is necessary to limit the impact of censoring, and allow smooth estimation with Machine Learning methods. Further limiting the impact of censoring, we use the 7-day centered rolling average of daily flows.

Although FB data has high coverage, FB users are not a random sample of the population. This raises concerns about representativeness. Figure A.1 in Appendix mitigates these concerns by showing a strong association between daily movements of FB users and the (estimated) number of daily border crossings in the pre-Covid-19 period, which are presented in Appendix Table A.1.^5^ However [19] shows that FB users are over-represented in the population aged 20-40, with a high level of education and an above-average income level. It means that groups of individuals under 20 or over 65 and those with lower income/education levels are under-represented in FB data. Although people under 20 and over 65 form a tiny minority of the population under investigation (i.e., commuters and business travelers), this is a limitation of our work. FB users are more likely to belong to the richest and healthiest parts of the movers’ population.

Let us denote by $M_{i\rightarrow jt}^{F}$ the count of FB movers from country *i* to country *j* at day *t*. When focusing on contiguous countries (i.e., the pairs of countries that exhibit the largest numbers of daily cross-border movements by far, and that are the least affected by censoring rules), the number of movers from *i* to *j* is almost identical to the number of movers from *j* to *i* at each day *t* (i.e., $M_{i\rightarrow jt}^{F} \simeq M_{j\rightarrow it}^{F}$). The reason is that border crossings predominantly consist of back-and-forth movements of commuting workers and business travelers, who move for short periods and for economic reasons^6^. This is also the case in the summer vacation period when considering a 7-day centered rolling average of daily flows. This means that $M_{i\rightarrow jt}^{F}$ and $M_{j\rightarrow it}^{F}$ are reflecting the same reality, and say nothing about the primary direction of the flows. For this reason, we define the level of *bilateral traffic* of FB users between countries *i* and *j* as:

1 $$ T_{ijt}^{F} \equiv \text{Max}\left[ M_{i\rightarrow jt}^{F},M_{j\rightarrow it}^{F}\right],   $$

and see it as a proxy for the scaled sum of the two unobserved unidirectional flows between the two countries, *ϕ*(*M*_*i*→*jt*_+*M*_*j*→*it*_), where the scale factor *ϕ* denotes the fraction of FB users in the actual number of movers (denoted by *M*_*i*→*jt*_ and *M*_*j*→*it*_). Using the maximum as weighting scheme has the advantage to limit the impact of censoring in the case movers in one of the two directions are below the threshold. As $T_{ijt}^{F}=T_{jit}^{F}$, we can get rid of the dyadic dimension of the data, treat each country pair as a one-dimensional observation, and divide the size of the sample by two. In the methodological section, however, we explain how priors about the primary direction of the flows can be used to improve the quality of fit of our models.

To avoid dealing with re-scaling issues, we express traffic counts as relative deviations from their initial or pre-Covid-19 levels – in our case, the levels observed at the outset of the pandemic (denoted by day 0). We thus use the relative deviation in bilateral traffic between day 0 and day *t*, $\tau _{ijt} \equiv \frac {T_{ijt}-T_{ij0}}{T_{ij0}}$, as a variable of interest instead of focusing on the level of traffic *T*_*ijt*_ itself. Modelling relative deviations is also helpful to avoid over-fitting large corridors at the expense of small corridors, and mitigates representativeness issues even if the scale factor (*ϕ*) varies across country pairs.

**Fig. 1:**
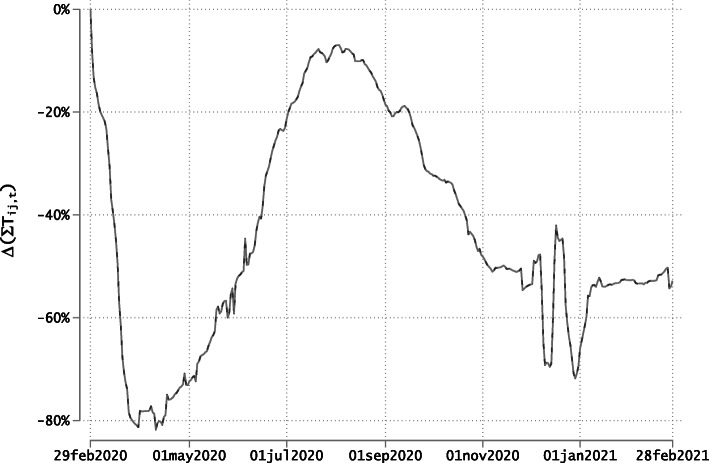
Aggregate Traffic Deviations from Pre-Covid Levels. Source: Facebook data on daily border crossings. Notes: Y-axis represents the average of *τ*_*ijt*_, the percentage change (times 100) in the 7-day moving average traffic compared to *t*=0 over all destinations. The weights are the traffic levels observed in pre-Covid-19 period (i.e., *t*=0)

Figure 1 portrays these relative deviations in the aggregate level of traffic between all country pairs included in our sample. The curve largely mirrors the three phases of the pandemic, depicting a stark drop in traffic in March 2020, a recovery during the spring and summer periods, and a new contraction in the post-summer period. Between end of February and early April 2020, the aggregate traffic level decreased from 720,000 to 130,000, implying a 82% drop. Aggregate traffic never fully recovered to the February levels in our period of observation. This also holds true during the summer vacation period when international travels were largely liberalized. The pace and strength of these changes vary across the three phases of the pandemic. The drop in March 2020 was strong and sudden, while the summer peak and the post-summer contraction were more gradual.

**Fig. 2:**
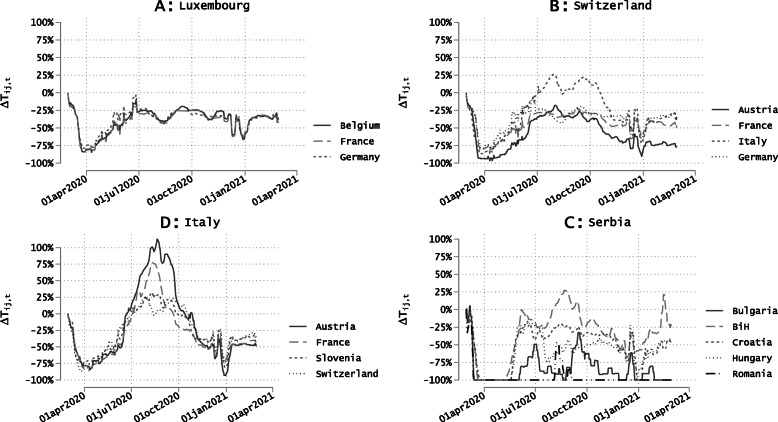
Traffic Deviations from Pre-Covid Levels for Selected Corridors. Source: Facebook data on daily border crossings. Notes: Y-axis represents *τ*_*ijt*_, i.e. percentage change (times 100) in corridor 7-day moving average traffic compared to *t*=0 in corridor *ij*

Aggregate fluctuations mask large differences across country pairs. Bilateral traffic returned to its pre-Covid level in a minority of cases. For the majority of corridors, however, the traffic level has not fully recovered. This is illustrated in Fig. 2, which depicts the evolution of people’s traffic in corridors involving four open countries, namely Luxembourg, Switzerland, Italy, and Serbia. Luxembourg is the country with the highest share of cross-border workers in Europe. Given the economy’s high reliance on cross-border workers, the Luxembourg government has never implemented international travel restrictions during the pandemic. Luxembourg experienced a significant drop in traffic in March 2020, whatever the partner country. After one month of lockdown, traffic levels recovered pretty quickly until reaching a plateau at about -25% since June 2020. Switzerland is the country with the largest number of cross-border commuters in Europe. This country experienced a larger drop during the first lockdown, and a slower recovery. Furthermore, the variability across corridors is considerably greater than in Luxembourg.

Italy has been severely impacted by the pandemic, and responded with national and international travel bans. We observe similar patterns of contraction and recovery during the first two quarters of 2020, followed by a substantial increase in traffic during the holiday summer period, and a second lockdown-type contraction in the post-Summer period. Finally, the patterns observed in Serbia are less conclusive as they are more severely affected by censoring rules. Serbia is an important origin and transit country for migrants and refugees entering the EU. Overall these patterns illustrate the need to account for corridor-specific heterogeneity when analyzing the determinants of bilateral traffic. Variations are likely to be influenced by seasonal effects, epidemiological risks, and policy measures implemented in the countries. We now turn to the description of the data sources used to proxy epidemiological conditions and the stringency of national policies.

### Explanatory features

We link variations in cross-border traffic during the pandemic to daily changes in epidemiological conditions and containment policies in the countries involved. We proxy the severity of the pandemic with the daily numbers of new Covid-19 cases and new Covid-19 related deaths in each country using data from [20]. With regard to containment measures, we use data on daily policy responses from the Oxford Covid-19 Government Response Tracker (OxCGRT) [21]. The latter database consists of 18 ordinal indicators capturing the levels of nonpharmaceutical interventions (NPIs). Based on our priors as to which policies likely affect mobility, we choose to include the eight mobility-related measures that form the “containment and closure policies” block (denoted by C1-C8 in the database) as well as proxies for the intensity of testing and contact tracing (denoted by H2 and H3). We rescale all sets of predictors between 0 and 1, and align them with the definition of the outcome variable using the centered 7-day rolling average at each day.

**Fig. 3:**
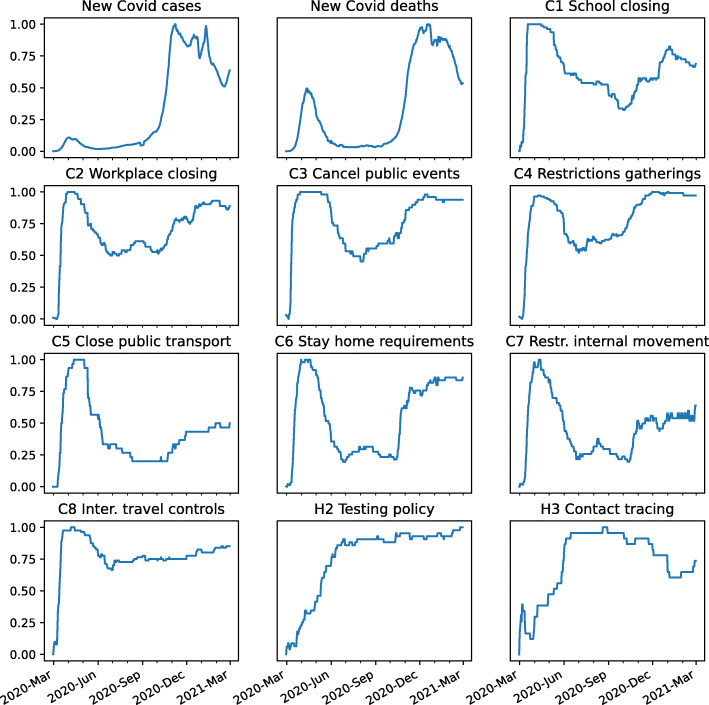
Evolution of the average value of Covid cases and government policy measures. Source: Oxford Covid-19 Government Response Tracker (OxCGRT). Note: The values of each indicator is scaled between 0 and 1, and the average is computed using the 30 countries included in our sample

For all features and days, Fig. 3 displays the cross-country mean level of each containment index over time. Values close to one represent higher Covid-19 cases/deaths or more stringent responses. As containment policies were implemented in most countries during the second half of March 2020, maximum values are observed during this period. Testing and contract tracing were implemented more heterogeneously across countries and peaked in the summer of 2020. Reported number of new Covid-19 related cases/deaths were much greater during the second wave and peaked at the end of the year 2020.^7^

**Fig. 4:**
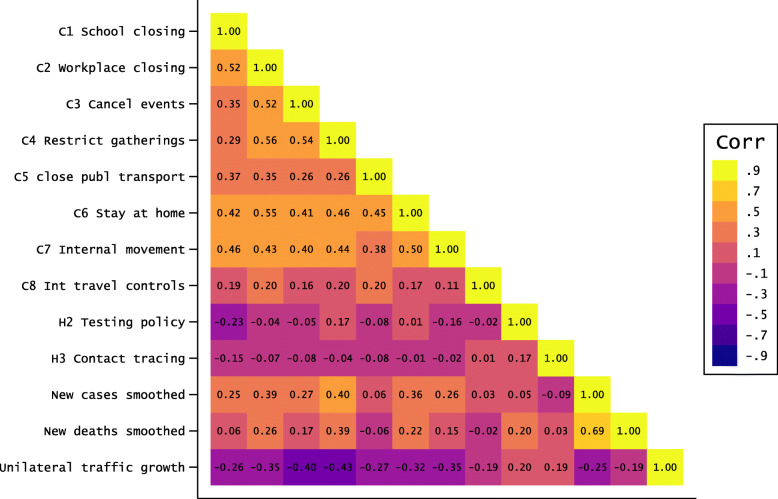
Correlation Matrix of the different variables. Source: Own computations. Notes: Unilateral traffic growth for each country *i* is the relative deviation in aggregate traffic involving country *i*, $\sum _{j=1}^{n} T_{ijt}$, as compared to the pre-Covid-19 period (*t*=0)

Figure 4 shows the cross-country correlations between each of the explanatory variables and the relative deviations in average traffic (i.e. deviation of the country-specific mean level of traffic with all potential partner countries in the sample). Containment policies are positively correlated with each other, and moderately correlated with epidemiological conditions, which allows us to include both sets of variables jointly in our regression and machine learning models. However, the fact that containment policies are correlated with each other raises concerns of multicollinearity, and motivates the usage of a limited number of synthetic policy indices. These indices are obtained by conducting a Principal Component Analysis (PCA) of all policy measures (C1-C8, and H2-H3) over the entire sample of observations, and by extracting the first two components. The first component mainly represents the C1-C8 measures which are strongly correlated with each other while the second component corresponds to a higher variance for the H2-H3 measures. We will compare the results obtained when using a comprehensive specification, including all (collinear) explanatory variables, with those obtained when using a parsimonious specification, including the two synthetic PCA components as predictors.

Turning to the main correlations of interest (i.e., correlations between the relative deviations in cross-border traffic and each predictor), the figure displays a moderate negative correlation with containment policies, as well as low and positive correlations with testing and tracing policies. The strongest associations are obtained for cancellations of public events, restrictions on public gatherings, requirements to stay at home, and restrictions on internal movements. Two interesting observations arise from these partial correlations. First, the negative correlation between measures of epidemiological intensity (Covid-19 cases and deaths) and changes in total traffic are rather low. This might suggest that, in contrast with studies on within-border mobility, the fear of being infected might play a less important role in explaining changes in cross-border movements. Second, among containment policies, the implementation of international travel bans is far from being the most strongly correlated covariate. This suggests that travel bans might be effective in limiting non-essential travels, but less effective to limit labor commuting flows and business travels, which represent the overwhelming majority of daily border crossings between European countries (see Table A.1). By contrast, cancellations of events and constraints on internal movements and gatherings are highly correlated with traffic variations, possibly because such constraints better proxy changes in economic activity and incentives to move. In the following section, we describe the regression and machine learning methods used to test these hypotheses using bilateral traffic growth as a dependent variable.

## Methods

Mobility patterns identified in the previous section might result from various factors such as travel bans, sanitary measures influencing economic costs and incentives to move for work and business (e.g. sectoral lockdown, work-from-home practices) or for leisure (e.g. shops, restaurant and bar closings), or the fear of the virus itself. Our goal here is to identify the determinants of the relative deviation in daily traffic of people between country *i* and country *j* (*τ*_*ijt*_), considering all NPIs and epidemiological daily indicators (*x*_*it*_ and *x*_*jt*_) during the Covid-19 crisis. Our models are also used to predict the effects of epidemiological restrictions, NPIs and mobility restrictions on traffic counts.

We combine two analytical methods, Econometric Modelling (EM) and Machine Learning (ML). EM and ML techniques are generally used for different purposes. In EM, gravity models are used to explain human mobility flows between two countries.^8^ EM models require imposing one analytical specification for the response function, which governs the derivatives of the dependent with respect to covariates. In the gravity specification, a large set of fixed effects are included to capture relevant confounders, and allow for identification of potential causal effects based on the so-called within variation. ML techniques are at the other extreme of the bias-variance tradeoff. They do not require strong analytical assumptions and allow, by design, to explore a larger set of regression functions including linear or polynomial combinations of the covariates. This increase of the so-called model *capacity* comes with two drawbacks. First, the models are more complex and are usually not easy to interpret. Contrary to EM, ML techniques might computationally suffer from the inclusion of large sets of control fixed effects. Second, there is always a risk of overfitting the training data and the identification of causation links is usually not an objective per se. Despite these difficulties, we use both EM and ML to identify converging messages, and the triangulation of results can serve to strengthen our evidence base.

We use four models exploring a broad range of learning techniques: (i) A gravity model based on the linear regression method [23]; (ii) A K-nearest neighbors method (KNN), which predicts the dependent variable by interpolation of its nearest observation neighbors in the training set [24]; (iii) A Gradient Boosting method (GBoost), whose predictions are based on a set of decision tree models [25]; (iv) A Multi-layer Perceptron (MLP), which is a classic neural network approach [26, 27]. The last three models rely on different ML regressors, each based on a distinct type of technique. We assess the predictive performance of each model using the very same (and standard) cross-validation ML methodology. The goal of the study is not to design a forecasting model, but rather to identify the main determinants of mobility, and to investigate whether these different approaches generate converging findings. Therefore, instead of the validating our model on a particular sub-period (as is usually done to evaluate a time-series model), the observations composing the cross-validation folds are randomly chosen within the full sample. All models are implemented in Python via the Skicit-learn library [28].

### Approaches with or without directional priors

Ideally, mobility models aim to characterize the evolution of the unidirectional flow of people (*M*_*i*→*jt*_) from an origin country *i* to a destination country *j* at day *t*, or of their relative deviation from the initial reference period $\left (\mu _{i\rightarrow jt} \equiv \frac {M_{i\rightarrow jt}-M_{i \rightarrow j0}}{M_{i\rightarrow j0}}\right)$, based on a set of features available for the same time period. Without loss of generality, the general functional form *f*_*M*_ of such a model can be written as:

2 $$ \mu_{i\rightarrow jt} = f_{M}(x_{it},x_{jt}, d_{ij}, d_{t}) + \eta_{i\rightarrow jt}   $$

where *x*_*it*_ represents the set of origin-specific determinant, *x*_*jt*_ is a set of destination-specific determinants, *d*_*ij*_ is a set of bilateral dummies capturing time-invariant bilateral resistance (including initial *M*_*i*→*j*0_, distance, language proximity, cultural proximity, etc.), *d*_*t*_ a set of day dummies capturing weekdays and seasonal trends (e.g. holiday season, general feeling of risk when traveling, etc.), and *η*_*i*→*jt*_ an error term. In our case, the vectors of explanatory variables *x*_*it*_ and *x*_*jt*_ capture the set of NPIs and epidemiological variables, and should also be interpreted as variations from period 0 since *x*_*i*0_ and *x*_*j*0_ are equal to zero in the pre-Covid-19 period.

With FB data, the primary direction of the cross-border flows is unknown, which implies that *M*_*i*→*jt*_ and *M*_*j*→*it*_ cannot be distinguished a priori. Instead, we observe the relative deviation in bilateral traffic, *τ*_*ijt*_, and we have to estimate the function *f*_*T*_ linking bilateral traffic to the set of explanatory features without being able to distinguish between origin- and destination-specific determinants. A learning approach without directional priors writes as:

3 $$ \tau_{ijt} = f_{T}(x_{it},x_{jt}, d_{ij}, d_{t}) + \eta_{ijt}   $$

It is possible, however, to discipline the model with priors about the direction of the flows. As bilateral traffic is a proxy for the sum of unidirectional flows (*T*_*ijt*_≃*ϕ*(*M*_*i*→*jt*_+*M*_*j*→*it*_)), relative deviations in *T*_*ijt*_ can be expressed as a weighted sum of the relative deviations in unidirectional flows: *τ*_*ijt*_=*ω*_*i*→*j*,0_×*μ*_*i*→*jt*_+*ω*_*j*→*i*,0_×*μ*_*j*→*it*_, where *ω*_*i*→*j*,0_=1−*ω*_*j*→*i*,0_ is the pre-Covid-19 share of unidirectional cross-border flows from country *i* to country *j* in total traffic between the two countries. Estimates for *ω*_*i*→*j*,0_ are constructed using pre-Covid-19 data on commuters, air travels and international migration, and then used as priors to discipline the model (these shares are depicted in Figure A.2 in Appendix).

We can thus create two sets of weighted features, namely $X_{ijt}^{o}$ for origin-specific effects, and $X_{ijt}^{d}$ for destination-specific effects, defined as follows:

$$\begin{array}{*{20}l} X_{ijt}^{o} = \omega_{i\rightarrow j0}x_{it}+\omega_{j\rightarrow i0}x_{jt}\\ X_{ijt}^{d} = \omega_{i\rightarrow j0}x_{jt}+\omega_{j\rightarrow i0}x_{it}.  \end{array} $$

The model with directional priors is obtained after replacing (*x*_*it*_,*x*_*jt*_) in Eq. (3) by $\left (X_{ijt}^{o},X_{ijt}^{d}\right)$. It writes as:

4 $$ \tau_{ijt} = f_{T}\left(X_{ijt}^{o},X_{ijt}^{d},d_{ij}, d_{t}\right) + \eta^{od}_{ijt}.   $$

If the true model for unidirectional flow (*f*_*M*_(.)) was linear, plugging weighted covariates in the estimated model for bilateral traffic (*f*_*T*_(.)) would allow retrieving the true origin- and destination-specific coefficients of interest accurately. Although this is not the case when *f*_*M*_(.) is non linear, using weighted covariates might improve the quality of fit or facilitate the interpretation of the results. The rationale is that the effect of policies depends on where they are implemented and on the primary direction of the flows. Suppose *ω*_*i*→*j*0_≃1 (i.e., flows mostly go from *i* to *j*), then an increase in restrictions/stringency at destination (resp. at origin) makes $X_{ijt}^{d}$ positive (resp. $X_{ijt}^{o}$ positive) and is more (resp. less) likely to reduce the flow of cross-border movements. Using directional priors allows approximating origin- and destination-specific effects without observing the direction of the flows during the pandemic year.

### Permutation feature importance

To identify the main causes of daily mobility variations, the importance of each feature is computed for the different regression approaches with *permutation feature importance* [29]. It has a the advantage of working similarly for all regression models considering them as a black-box models [30]. It is defined to be the decrease in the regression score when a single feature value is randomly shuffled across observations. More exactly, for a given model *f*, it first calculates a baseline score *S*_*f*_ provided by *f* when it is fitted, and then evaluated with a certain metric on the whole sample. Then for each possible feature *x* the modified score $S_{f,x}^{*}$ is computed by evaluating *f* on the transformed data set where the values of feature *x* are randomly permuted across all observations. The mean importance of the feature *x* for the model *f* is computed as:

5 $$ I_{f,x} = \frac{1}{K} \sum_{k=1}^{K} \frac{S_{f}-S_{f,x,k}^{*}}{S_{f}}  $$

where *K* is the number of random permutations realized for each feature, and $S_{f,x,k}^{*}$ is the $S_{f,x}^{*}$ score for the *k*^*th*^ permutation.

In order to compare the importance values of the different models in an equivalent manner, the values *I*_*f*,*x*_ are scaled between 0% and 100% separately for each model *f*. The mean features importance *I*_*f*,*x*_ are computed over 10 permutations using the negative mean absolute error (MAE) and the Root Mean Squared Error (RMSE). This means that the bigger *I*_*f*,*x*_, the more permutations of the feature *x* degrades the quality of predictions for the model *f* and the feature is considered as more importantly associated with the target variable.

## Results

We present our results in two steps. First, we assess the predictive power of the various models. This implies comparing learning methods with or without directional priors and with or without day/corridor control dummies. We compare their predictive performance by using out-of-sample predictions and computing the MAE and RMSE. Second, we use the estimated models to rank the importance of different features relying on permutation techniques. Using multiple models allows assessing the robustness of our findings.

### Validation of models

We first investigate whether adding priors about the direction of the flows and/or adding a full bunch of day and corridor dummies improves the performance of our learning models. Models without directional priors are described in Eq. (3), while models with priors use weighted regressors, as described in Eq. (4). A 10-fold cross-validation over the whole data set (from the 29^th^ February 2020 until the 28^th^ February 2021) is realized for each model to assess its performance. Table 1 reports the MAE, RMSE and their standard error across cross-validation folds obtained with different learning methods.

**Table 1:** MAE comparison of the different models with or without directional priors and dummies

	Linear	KNN	G-Boost	MLP	Linear	KNN	G-Boost	MLP
	**Panel A: No dummies - No prior**				**Panel B: No dummies - Priors**			
avg MAE	0.194	0.018	0.073	0.051	0.201	0.019	0.042	0.057
std MAE	(0.009)	(0.001)	(0.001)	(0.005)	(0.005)	(0.001)	(0.001)	(0.003)
avg RMSE	0.285	0.042	0.106	0.083	0.287	0.043	0.064	0.089
std RMSE	(0.017)	(0.009)	(0.003)	(0.011)	(0.009)	(0.005)	(0.002)	(0.008)
	**Panel C: Dummies - No prior**				**Panel D: Dummies - Priors**			
avg MAE	0.135	0.020	0.050	0.041	0.134	0.020	0.049	0.038
std MAE	(0.005)	(0.001)	(0.002)	(0.003)	(0.005)	(0.001)	(0.001)	(0.003)
avg RMSE	0.210	0.045	0.081	0.068	0.203	0.047	0.077	0.064
std RMSE	(0.010)	(0.006)	(0.006)	(0.006)	(0.009)	(0.005)	(0.002)	(0.005)

Note: The table compares the performances of the 4 different approaches (Linear, KNN, G-Boost and MLP) with and without directional priors (*ω*_*i*→*j*,0_), and with or without day/corridor dummies (*d*_*t*_ and *d*_*ij*_). Errors are computed from a 10-fold cross-validation on the whole sample

It shows that directional priors (*ω*_*i*→*j*,0_ and *ω*_*j*→*i*,0_) do not bring significant additional predictive power under most learning approaches when day and corridor dummies are not factored in (see Panels A and B). The only exception is the G-Boost method. On the contrary, when day and corridor dummies are included (Panels C and D), adding directional priors slightly improve the quality of fit with virtually all learning techniques (except with KNN). In addition, we show below that distinguishing between origin- and destination-specific effects as in Eq. (4) makes the interpretation of the results much easier. Therefore, the model with directional priors will be prioritized in the rest of the analysis.

Second, we investigate whether the inclusion of day-specific effects – i.e., 366 time dummies, *d*_*t*_, that are common to all corridors and capture unobserved variations such as seasonal changes, synchronized fears of infection, etc. – and corridor-specific effects – i.e., 45 corridor dummies, *d*_*ij*_, that are time invariant and capture unobserved variations such as the skill level of the cross-border workforce, linguistic and cultural proximity between countries, etc. – improves the predictive power of our models. Again, we perform another 10-fold cross-validation on different versions of each model. Panels C and D in Table 1 includes both sets of dummies jointly, with or without directional priors. In the absence of directional priors, the inclusion of day and corridor dummies reduces the MAE and RMSE by 20 to 30% whatever the learning technique used. When directional priors are factored in, the dummies improve the performance of the linear and MLP models, whereas they deteriorate the quality of fit under the KNN and G-Boost models. This is because adding day and corridors dummies drastically increases the number of parameters to be estimated, and some ML approaches (like KNN) are known to suffer from the curse of dimensionality.

To further explore this issue, Table A.2 in Appendix considers the model with directional priors and adds one set of dummies at a time. The inclusion of 45 corridor-specific dummies always improves the quality of fit. On the contrary, the inclusion of 366 day-specific dummies deteriorates the performance of KNN and G-Boost methods. This confirms that the gains from adding information about unobserved common time trends, which might already be captured by the relatively well synchronized trends in observed epidemiological conditions and containment measures, is outbalanced by the costs linked to the inflated dimensionality of the computation problem.

Third, ML techniques always outperform the linear EM model. This result was also expected given that ML is based on more complex prediction methods that allow for non-linear relationships between variables, and account for non-stationary variations contained in the matrices of $X_{ijt}^{o}$ and $X_{ijt}^{d}$. The KNN always produces the best quality of fit. The error of this approach is minimal when the number of neighbors *k* used to estimate the relative deviations in traffic is low (say, 2 or 3). Its impressive performance in 10-fold cross-validation can be explained by the fact that the model finds a small number of observations for which the relative deviations in traffic are similar to those that must be predicted. In general, the closest neighbors are observations of the days preceding or following the daily level of traffic observed in the same corridor.

### Main sources of variations in cross-border mobility in COVID times

In order to identify the government policy indicators having the greatest impact on relative deviations in traffic, the importance of each feature is computed for the different approaches involving directional priors and dummies. Directional priors allow us to distinguish the effects of origin-specific features from those of destination-specific features. Table 2 presents the results from this exercise. Features are ranked by decreasing order on the basis of the average predictive power across the four learning techniques. The column ‘Avg.’ gives the mean value of error metric averaged over the four models. Panel A provides the results obtained with the saturated models including the large set of corridor and day dummies, which is the first-best model when using linear and MLP learning techniques. Panel B gives the results obtained with corridor dummies only, which is the first-best model when using the KNN and G-Boost techniques. Results of Panel B will be discussed in the next section. In the top part of the table, the models use all individual features depicted in Fig. 3, despite the high level of correlation between some of them. In the bottom part of the table, the models use synthetic containment features derived from a PCA analysis.

**Table 2:** Feature ranking by origin and destination

	Panel A					Panel B
	Corridor & Day dummies					Corr. dum.
Features	Linear	KNN	G-Boost	MLP	Avg.	Avg.
Indiv. features						
Origin - C1 School closures	100	69	100	59	82	85
Destin - C1 School closures	94	60	36	85	68	68
Destin - C3 Cancel public events	19	64	77	68	57	57
Origin - C3 Cancel public events	10	100	33	65	52	46
Origin - C7 Restr. Internal movement	0	93	16	100	52	51
Origin - H2 Testing policy	14	7	87	92	50	43
Origin - C4 Restrictions gatherings	50	51	40	54	49	44
Origin - C6 Stay home requirements	92	16	12	59	45	21
Destin - C6 Stay home requirements	17	15	96	54	45	45
Destin - C4 Restrictions gatherings	6	48	70	25	37	42
Destin - C7 Restr. Internal movement	13	66	12	58	37	29
Origin - H3 Contact tracing	5	12	27	44	22	22
*Origin - C8 International travel bans*	1	1	45	40	22	18
Origin - New Covid deaths	0	7	73	5	21	46
Origin - New Covid cases	11	0	21	52	21	75
**Destin - New Covid deaths**	6	10	64	0	20	49
Destin - C5 Close public transport	34	6	1	39	20	9
Destin - H3 Contact tracing	31	21	2	14	17	7
Destin - C2 Workplace closing	5	33	7	23	17	21
*Destin - C8 International travel bans*	0	12	20	32	16	24
**Destin - New Covid cases**	12	0	43	6	15	38
Origin - C5 Close public transport	18	7	0	31	14	12
Origin - C2 Workplace closing	1	23	10	14	12	23
Destin - H2 Testing policy	9	0	2	10	4	6
Synthetic features						
Destin - Component 1	100	100	100	100	100	100
Origin - Component 1	13	75	20	49	39	48
Origin - Component 2	1	55	17	19	23	26
Destin - Component 2	9	50	0	0	15	17
Origin - New Covid cases	0	0	18	30	12	75
**Destin - New Covid deaths**	5	14	6	11	9	49
Origin - New Covid deaths	1	13	3	9	6	46
**Destin - New Covid cases**	0	2	1	6	2	38

Notes: The different features are ranked following the permutation importance method. For each approach, we provide results obtained with the model including day/corridor dummies (cols. 1-5) and the version including corridors dummies only (col. 6). Directional priors are always used to identify the effects of origin- and destination-specific features. The importance values of each feature is computed over 10 permutations using the negative mean absolute error (MAE). The resulted values are scaled between 0% and 100% separately for each model. The col. ‘Avg.’ averages the results obtained with the four learning techniques. The features are ranked according to the average importance of the models including the day/corridor dummies (Panel A). In Panel B, we only report the ‘Avg.’ score without reporting the model-specific results

When considering all individual features, the ranking based on their predictive power varies across models. We identify, however, several common and interesting findings. *First*, school closures in the origin country has the largest average impact on the variation in daily traffic. Remember that cross-border traffic predominantly consists of labor commuting flows and business travels. School closures at origin imply that many parent workers are forced to take parental leave and cannot commute to work. In the same vein, school closures in the destination country are also paralyzing economic activity in the destination country and reduce incentives to move. This result is in line with [31], who finds that school closures were among the most important predictors of internal mobility in March 2020. *Second*, variations in traffic are mostly impacted by containment measures. Based on the average predictive power (col. ‘Avg.’), ten out of the twelve most predictive features involve C-type containment measures implemented in the origin or destination countries. *Third*, the fear of being infected in the destination country, as proxied by the destination-specific number of Covid-19 deaths and cases (appearing in bold characters in Table 2) are among the least predictive features. *Fourth*, international travel bans in origin and destination countries (appearing in italics in Table 2) also have a low predictive power. We thus conclude that cross-border daily flows are marginally influenced by the fear of infection and international travel bans. They are mostly governed by the stringency of internal containment policies and by family constraints. It is worth noticing that models without directional priors deliver very similar results, as illustrated in Table A.3 in the Appendix.

In addition to school closures, the top panel of Table 2 suggests that the most important containment measures are the cancellation of public events, restrictions on gatherings, restrictions on internal movements and stay-home requirements. In addition, the twelve most predictive features include 5 destination-specific and 7 origin-specific measures. However, as illustrated in Fig. 4, these measures are highly correlated at the national level. Hence, instead of feeding the model with correlated features, the bottom part of the table uses synthetic indices of containment and sanitary measures. We use a PCA analysis to reduce the dimensionality of the origin- and destination-specific containment measures and we extract the first two components of the PCA.

Remember that the first PCA component can be interpreted as an average index of stringency of containment measures (i.e. C1-C8 indices); the second component captures testing and tracing policies (i.e. H2 and H3). The results clearly reveal that the stringency of containment measures in the destination country has, by far, the greatest predictive power. The average stringency of containment measures at origin is the second most predictive power, with an average importance equal to 40% of that of the destination country. This comforts the idea that cross-border daily flows of people mostly involve economic/essential movements which can only be influenced by changes in incentives to move or coercive mobility constraints. In line with the top part of the table, variables influencing the fear of infection have negligible impacts on border crossings.

## Discussion

The EM and ML techniques used in this paper allow highlighting a strong association between the evolution of bilateral traffic between contiguous countries and containment policies in the destination country as well as school closures in the origin country. Association does not imply causation. It could be argued that this statistical association is governed by the influence of unobserved characteristics affecting both policy changes and mobility simultaneously, or that a reverse causation mechanism operates (i.e. cross-border mobility influences policy reforms). The fact that our results are robust to the inclusion of a large set of day and corridor dummies capturing unobserved time- and corridor-specific characteristics strongly mitigates the first misspecification concern.

With regard to reverse causation, concerns are mitigated by the use of high-frequency data. We cannot reject the possibility of a mobility-driven propagation of the virus requiring new containment measures. However, such a mechanism takes time to operate. Mobility shocks at day *t* do not generate immediate and visible epidemiological consequences, and policy responses are also implemented with a certain delay. By contrast, our estimates suggest that containment policies are immediately associated with changes in cross-border mobility. This prudently supports the existence of a causation effect of containment measures on mobility.

An opposite argument that goes against the reverse causation issue is that it also takes time for information about epidemiological conditions to be assimilated by potential movers. Hence, the fear of infection could be better proxied by the lagged numbers of Covid-19 cases and deaths. Our results are preserved and even reinforced when using lagged proxies for the fear of infection. More precisely, traffic at day *t* is very badly predicted by the number Covid-19 cases and deaths observed one or two weeks before the date (see Table A.4 in the Appendix). Hence, we can reasonably rule out that the low impact of infection fears at destination is driven by a misspecification problem.

In the same vein, it could be argued that fears are strongly synchronized across countries and captured by the day dummies. These concerns are mitigated by the fact that removing the day-specific dummies does not alter our conclusions. In Panel B of Table 2, day dummies are excluded. The only significant change is that the number of Covid cases in the origin country has a greater predictive power, possibly implying that people in sick leave self-isolate and stop moving. However, the predictive power of international travel bans (in italics) and fears to be infected at destination (epidemiological conditions at destination are in bold characters) remain low when using both individual and synthetic features. Again, the stringency of containment measures in the destination country has the greatest predictive power by far.

**Table 3:** Estimates of the average count of daily cross-border outflows in 2019

	Daily count estimates					As percent of Total		
	Commuters	Air pass.	Migrants	Total		Commuters	Air pass.	Migrants
AUT	159678	169395	441	329514		0.484	0.514	0.001
BEL	133742	241103	729	375574		0.356	0.641	0.001
DNK	44732	45719	77	90528		0.494	0.505	0.000
FIN	22532	21311	195	44038		0.511	0.483	0.004
FRA	396457	417902	670	815029		0.486	0.512	0.000
DEU	472160	361659	2770	836589		0.564	0.432	0.003
GRC	1714	24700	4	26418		0.064	0.934	0.000
IRL	23892	108689	61	132642		0.18	0.819	0.000
ITA	139103	115605	952	255660		0.544	0.452	0.003
LUX	152407	4829	76	157312		0.968	0.030	0.000
NLD	102000	267917	296	370213		0.275	0.723	0.000
PRT	55785	87144	157	143086		0.389	0.609	0.001
ESP	79857	346395	36	426288		0.187	0.812	0.000
SWE	51000	87565	91	138656		0.367	0.631	0.000
GBR	76300	465893	873	543066		0.140	0.857	0.001
EU15	1911359	2765826	7428	4684613		0.408	0.590	0.001
BIH	17017	21296	0	38313		0.444	0.555	0.000
BGR	52571	33019	42	85632		0.613	0.385	0.000
HRV	36557	28639	33	65229		0.560	0.439	0.000
CZE	122160	62294	156	184610		0.661	0.337	0.000
EST	10642	18674	6	29322		0.362	0.636	0.000
HUN	100196	62174	217	162587		0.616	0.382	0.001
LTU	2414	14435	0	16849		0.143	0.856	0.000
NOR	8232	50767	178	59177		0.139	0.857	0.003
POL	205314	135350	109	340773		0.602	0.397	0.000
ROU	65550	81892	207	147649		0.443	0.554	0.001

Sources: Numbers of daily commuters are extracted from Eurostat data by Nuts2 region in 2019; Numbers of air passengers are extracted from Eurostat monthly statistics on air passenger transport in February 2019; Data on international migrants are extrapolated from [34] for the year 2015, assuming a conservative 50% growth in the flows between 2015 and 2020

**Fig. 5:**
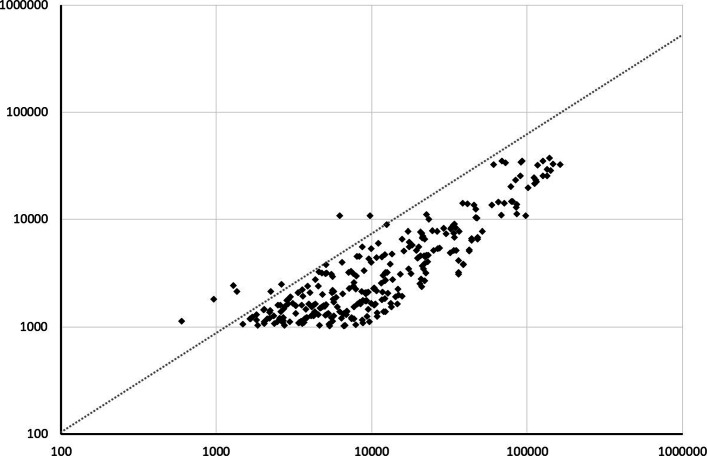
Daily traffic estimates (X-axis) and FB data (Y-axis) by corridor (1 March 2020). Sources: Numbers of daily commuters are extracted from Eurostat data by Nuts2 region in 2019; Numbers of air passengers are extracted from Eurostat monthly statistics on air passenger transport on March 1, 2020; Data on international migrants are extrapolated from [34] for the year 2015, assuming a conservative 50% growth in the flows between 2015 and 2020. FB data are the Facebook data on daily border crossings on March 1, 2020. Note: All variables are expressed in logs

Finally, one may fear that our results can be affected by the quality of data as well as their comparability across countries (see [32] for a discussion of the strengths and weaknesses of data sources on Covid-19). Data on Covid-19 threats and NPIs (such as the number of new cases and deaths) are likely to be subject to country and time biases due to multiple reasons (e.g. changes in testing strategies, seasonal effects, variations in reporting delays or in the classification of Covid-related deaths, etc.). It is worth emphasizing, however, that the *Permutation Feature Importance* technique that we use is, by construction, robust to noise.^9^ In addition, the systematic use of bilateral and time dummies (*d*_*ij*_ and *d*_*t*_) allows us to mitigate the biases caused by unobserved variations in the quality of data across country pairs and periods.

## Conclusion

Existing literature shows that people within-border mobility has drastically declined in times of Covid-19, primarily because of the fear to be infected in parts of the population. To the best of our knowledge, our study is the first to analyze the effect of Covid-19 and related containment measures on people’s cross-border movements. In line with the findings above, we also document a sharp decline in cross-border mobility in general, especially during the first lockdown and in the second and third waves of the pandemic. However, these variations in cross-border mobility are mostly induced by local containment policies in the destination country, and school closures in both countries. The fear of infection and international travel bans have little influence on cross-border movements.

The likely reason is that cross-border daily flows of people are predominantly made of commuting workers and business travelers who move for economic/essential reasons. These economic flows are observed between contiguous countries, and account for 99% of international movements of people when compared with the flows of migrants and refugees. Their magnitude varies with the economic costs and incentives of moving, which depend on lockdown measures and on the stringency of internal containment policies. In addition, international travel bans do not apply to commuters and businessmen. Although there is no consensus on the fact that these flows contribute to the propagation of the virus, policy-makers must be aware that economic movers hardly adapt their mobility decisions to epidemiological threats. Border crossings can only be controlled with internal coercive policies.

## Appendix A: Appendix – figures and tables

**Fig. 6:**
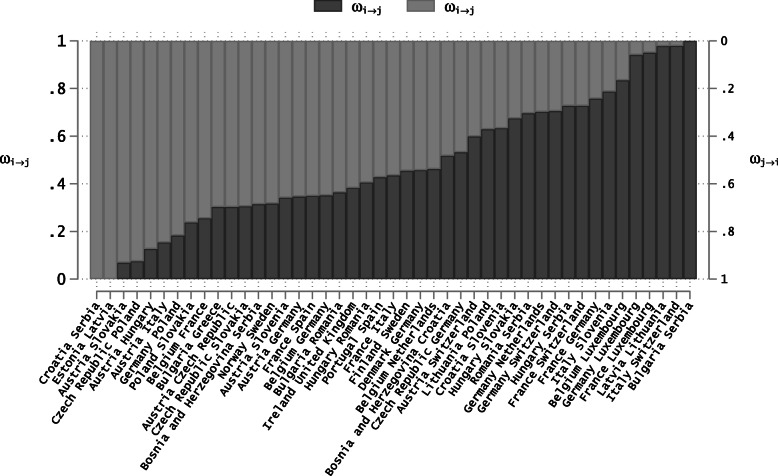
Distribution of Weights. Sources: Numbers of daily commuters are extracted from Eurostat data by Nuts2 region in 2019; Numbers of air passengers are extracted from Eurostat monthly statistics on air passenger transport on March 1, 2020; Data on international migrants are extrapolated from [34] for the year 2015, assuming a conservative 50% growth in the flows between 2015 and 2020. Notes: Countries on X-Axis are ordered as *country*_*i*_*country*_*j*_, thus linked to *ω*_*i*→*j*_. The opposite weight *ω*_*j*→*i*_=1−*ω*_*i*→*j*_ refers to the weight of *country*_*j*_*country*_*i*_

**Table 4:** MAE comparison of the different models with and without day/corridor dummies

	Linear	KNN	G-Boost	MLP	Linear	KNN	G-Boost	MLP
	**Panel A: No dummies**				**Panel B: Day dummies**			
avg MAE	0.201	0.019	0.042	0.057	0.179	0.181	0.056	0.059
std MAE	(0.005)	(0.001)	(0.001)	(0.003)	(0.004)	(0.005)	(0.001)	(0.003)
avg RMSE	0.287	0.043	0.064	0.089	0.259	0.269	0.084	0.090
std RMSE	(0.009)	(0.005)	(0.002)	(0.008)	(0.008)	(0.008)	(0.002)	(0.004)
	**Panel C: Corridor dummies**				**Panel D: Corridor & Day dummies**			
avg MAE	0.156	0.018	0.037	0.048	0.134	0.020	0.049	0.038
std MAE	(0.006)	(0.001)	(0.001)	(0.004)	(0.005)	(0.001)	(0.001)	(0.003)
avg RMSE	0.231	0.043	0.059	0.086	0.203	0.047	0.077	0.064
std RMSE	(0.010)	(0.005)	(0.002)	(0.009)	(0.009)	(0.005)	(0.002)	(0.005)

Notes: The table compares the performances of the 4 different approaches with and without the day- and corridor-specific dummies. All models are estimated with directional priors. Errors are computed from a 10-fold cross-validation on the whole data set

**Table 5:** Averaged feature ranking across models and specifications without directional priors

	Panel A: Corridor & Day dummies					Panel B: Corridor dummies				
Features	Linear	KNN	G-Boost	MLP	Avg.	Linear	KNN	G-Boost	MLP	Avg.
C1 School closures	100%	78%	98%	91%	93%	100%	100%	86%	86%	93%
C3 Cancel public events	14%	100%	79%	84%	69%	21%	100%	60%	76%	64%
C6 Stay home requirement	56%	18%	78%	71%	56%	25%	23%	64%	38%	38%
C7 Restr. Internal movement	6%	97%	20%	100%	56%	1%	84%	27%	100%	53%
C4 Restrict gatherings	28%	60%	80%	49%	54%	9%	76%	65%	64%	53%
H2 Testing policy	11%	4%	64%	64%	36%	26%	2%	49%	30%	36%
**New Covid deaths**	3%	10%	100%	0%	28%	0%	77%	100%	0%	44%
*C8 Inter. travel controls*	0%	8%	47%	44%	25%	3%	17%	44%	32%	24%
H3 Contact tracing	18%	20%	20%	35%	23%	12%	1%	19%	32%	16%
**New Covid cases**	11%	0%	46%	35%	23%	11%	76%	63%	87%	59%
C5 Close public transport	26%	7%	0%	43%	19%	15%	0%	0%	31%	11%
C2 Workplace closing	2%	34%	12%	21%	17%	2%	46%	23%	33%	26%

Notes: The different features are ranked following the permutation importance method. For each approach, we provide results obtained with the model including day/corridor dummies (cols. 1-5) and the version including corridors dummies only (cols. 6-10). Directional priors are not included. The importance values of each feature is computed over 10 permutations using the negative mean absolute error (MAE). The origin- and destination-specific features importance are aggregated by taking the mean between the 2. Finally, the resulted values are scaled between 0% and 100% separately for each model. The last column in each panel presents the mean value of importance averaged over the four models. The features are ranked according to the average importance of the models including the corridor and day dummies

**Table 6:** Feature ranking including lagged epidemiological conditions

	Panel A: Corridor & Day dummies					Panel B: Corridor dummies				
Features	Linear	KNN	G-Boost	MLP	Avg.	Linear	KNN	G-Boost	MLP	Avg.
C1 School closing	100%	69%	81%	68%	79%	100%	83%	79%	97%	90%
C3 Cancel public events	10%	100%	56%	78%	61%	19%	100%	58%	77%	63%
C7 Restr. Internal movement	5%	89%	22%	100%	54%	4%	74%	20%	100%	49%
C6 Stay home requirements	60%	20%	79%	55%	54%	27%	24%	74%	31%	39%
C4 Restrictions gatherings	12%	57%	100%	33%	51%	5%	74%	100%	32%	53%
H2 Testing policy	12%	6%	69%	54%	35%	20%	2%	67%	29%	30%
H3 Contact tracing	23%	15%	37%	29%	26%	21%	0%	34%	34%	22%
C5 Close public transport	25%	11%	7%	32%	19%	14%	5%	4%	33%	14%
C8 Inter. travel controls	0%	7%	30%	28%	16%	11%	16%	28%	21%	19%
C2 Workplace closing	0%	32%	8%	19%	15%	2%	41%	5%	12%	15%
New Covid cases *t*−7	12%	0%	8%	12%	8%	14%	34%	6%	46%	25%
New Covid deaths *t*	4%	12%	12%	0%	7%	10%	49%	13%	12%	21%
New Covid cases *t*	1%	1%	0%	24%	7%	4%	41%	0%	68%	28%
New Covid cases *t*−14	4%	1%	9%	8%	6%	6%	33%	8%	0%	12%
New Covid deaths *t*−14	0%	12%	6%	5%	6%	0%	53%	7%	21%	20%
New Covid deaths *t*−7	1%	10%	4%	3%	5%	8%	48%	4%	44%	26%

Notes: The different features are ranked following the permutation importance method. The importance values of each feature is computed over 10 permutations using the negative mean absolute error (MAE). Four new variables are inserted in addition to the ones includes in the regression: 7- and 14-days of new Covid cases and deaths are included. For each approach, we provide results obtained with the model including day/corridor dummies (cols. 1-5) and the version including corridors dummies only (cols. 6-10). Directional priors are not included. The origin- and destination-specific features importance are aggregated by taking the mean of the 2. Finally, the resulted values are scaled between 0% and 100% separately for each model. The last column in each panel presents the mean value of importance averaged over the four models. The features are ranked according to the average importance of the models including the corridor and day dummies

### A.1 Representativeness of facebook data

A critical question relates to the representativeness of FB data. On average, comparison between the number of FB users and population size by country reveals that the number of FB users with *location services enabled* represents 5.6% of the population in Europe, on average. However, this fraction is rather uniform across countries, which suggests that FB data might be representative of the population as a whole. This does not mean, however, that data on FB users are representative of the population of cross-border movers, as using internet connection or activating location services abroad can be costly [33]. To address this point, we produce estimates of the number of daily cross-border inflows in the pre-Covid period, and compare them with FB data. We collect data on commuting flows from the Labor Force Survey for 2019,^10^data on air passengers as of March 2019,^11^ and estimates of immigration flows from [34].^12^

Overall, Table A.1 shows that migration flows account for a tiny (say negligible) proportion of daily movements, implying that FB data mostly capture commuting flows and business travels, as well as holiday-maker’s moves in vacation periods. This implies that the largest flows are observed between contiguous countries and business partners. Combining corridors available in our sample and corridors involving non-EU countries, Figure A.1 shows that FB data as of the first week of March 2020 are highly correlated with estimates of actual flows in the year 2019, which suggests that FB data might also be representative of cross-border movements.

### A.2 Parameters of the mL methods

We describe in this section the parameters of the different ML models used in this paper. In order to tune the models, a boosting sample corresponding of 70% of the whole dataset is used (from the 29^th^ February 2020 until the 16^th^ November 2020). We use the following parameters:

- *K Nearest Neighbors.* The models uses 5 neighbors and it has the distance metric as weight function.
- *Gradient Boosting.* The Gradient Boosting model has least square regression as loss function, a learning rate of 0.1 and a number of estimators of 100 (which is equivalent to the number of boosting stages to perform).
- *Multi-Layer Perceptron.* The MLP Regressor has 2 hidden layers of 100 neurons each. The activation function for the layer is the rectified linear unit function. It uses an adam solver with minibatches of size 50.

### A.3 Additional figures and tables

Some additional figures and tables cited in the core of the text are found below.

## Data Availability

The datasets supporting the conclusions of this article are available in the Zenodo repository Cross-border-Mobility-Responses-to-Covid-19-in-Europe, 10.5281/zenodo.4719559

## Abbreviations

EM: Econometric Modelling
EU: European Union
FB: Facebook Inc.
G-Boost: Gradient Boosting
KNN: K-nearest neighbors method
MAE: Mean Absolute Error
ML: Machine Learning
MLP: Multi-layer Perceptron
NPIs: non-pharmaceutical interventions
OxCGRT: Oxford Covid-19 Response Tracker
PCA: Principal Component Analysis
RSME: Root Mean Squared Error

## Footnotes

1 We understand mobility as “the movement of human beings in space and time” [1]. This broad definition is in line with the mobility measure that we use in our quantitative analysis, as that includes all types of movement.

2 By big data, we mean extremely large data sets that may be analyzed computationally to reveal patterns, trends and associations, especially those related to human behaviors and interactions, according to the *Oxford Languages* (https://languages.oup.com/google-dictionary-en/).

3 The recent OECD migration outlook reveals that issuance of new visas and permits in OECD countries plummeted by 46% in the first half of 2020 (by 72% in the second quarter), as compared with the same period in 2019. However, international migrants and refugees account for a tiny proportion, not a say a negligible proportion, of daily cross-border movements of people between European countries. Daily flows predominantly consist of commuting workers and business travels.

4 A number of recent studies such as [17, 18] use data extracted from FB advertising API to identify migrants and model migration patterns. Note that our data source is different as we only have access to aggregate bilateral counts, without further details on movers’ characteristics. The advantage of our data source is, however, that it is available on a daily basis.

5 We also find a strong association between the number of FB users and population size at the regional level. Results are available upon request.

6 Note that we only observe the total number of movers within a day, and neither the duration of their stay nor the direction of the movement. The vast majority of movers cross the border twice as they return to their origin country within a day or so. This is why we model cross-border traffic and use a trick below to account for the presumed direction of the flows.

7 Epidemiological conditions are likely to subject to measurement errors. For example, testing and tracing practices played an important role in determining the number of detected cases.

8 Gravity models aim to predict bilateral flows between two entities based on their size, on their economic characteristics, and on the distance between them [22].

9 It could be further supplemented with a leave-one-out sensitivity analysis on countries [32].

10 Labor Force Surveys are provided by Eurostat. We use data on commuting by NUTS2 region, and allocate commuters aged 15 to 64 by destination on the basis of contiguity links with other countries. When a NUTS region shares several contiguity links, we share the number of commuters proportionately to the level of income per capita in the destination country. To identify daily movements, we assume that each commuter spend 4 days per week in the place of work, and multiply LFS number by 4/7.

11 We use monthly statistics on Air passengers from Eurostat by airport of departure, and allocate them by destination using FB data. To identify daily movements, we divide monthly flows by 30.

12 The latter are available until the year 2015; we multiply them by 1.5 to proxy the level in 2019. To identify daily movements, we divide annual inflows by 360.
